# Clinical outcomes of patients after nipple-sparing mastectomy and reconstruction based on the expander/implant technique

**DOI:** 10.1007/s00595-020-02175-4

**Published:** 2020-11-13

**Authors:** Uhi Toh, Miki Takenaka, Nobutaka Iwakuma, Yoshito Akagi

**Affiliations:** 1 Department of Surgery, Kurume University School of Medicine, 67 Asahi-machi, Kurume, 830-0011 Japan; 2 Department of Breast Surgery, National Hospital Organization Kyushu Medical Center, Fukuoka, Japan

**Keywords:** Breast cancer, Nipple-sparing mastectomy, Implant-based breast reconstruction

## Abstract

Advances in multi-modality treatments incorporating systemic chemotherapy, endocrine therapy, and radiotherapy for the management of breast cancer have resulted in a surgical-management paradigm change toward less-aggressive surgery that combines the use of breast-conserving or -reconstruction therapy as a new standard of care with a higher emphasis on cosmesis. The implementation of skin-sparing and nipple-sparing mastectomies (SSM, NSM) has been shown to be oncologically safe, and breast reconstructive surgery is being performed increasingly for patients with breast cancer. NSM and breast reconstruction can also be performed as prophylactic or risk-reduction surgery for women with BRCA gene mutations. Compared with conventional breast construction followed by total mastectomy (TM), NSM preserving the nipple–areolar complex (NAC) with breast reconstruction provides psychosocial and aesthetic benefits, thereby improving patients’ cosmetic appearance and body image. Implant-based breast reconstruction (IBBR) has been used worldwide following mastectomy as a safe and cost-effective method of breast reconstruction. We review the clinical evidence about immediate (one-stage) and delayed (two-stage) IBBR after NSM. Our results suggest that the postoperative complication rate may be higher after NSM followed by IBBR than after TM or SSM followed by IBBR.

## Introduction

Breast cancer is the most commonly diagnosed cancer in women worldwide. In Japan alone, breast cancer was diagnosed in nearly 10,000 patients in 2016 [1, 2]. The incidence of breast cancer is also increasing significantly in line with improved detection and screening techniques. Despite this increase, the recent 5-year survival rate of patients diagnosed with breast cancer in Japan was 92.7% [3]. Because of the evolving management and treatment of breast cancer using combinations of systemic chemotherapy, endocrine therapy, and radiotherapy, the standard surgical treatment for breast cancer has undergone a paradigm shift toward less-aggressive surgery combining breast-conserving or breast-reconstruction therapy for a higher emphasis on cosmesis [4, 5]. Compared with skin-sparing mastectomy (SSM) with or without nipple reconstruction, nipple-sparing mastectomy (NSM) preserving the nipple–areolar complex (NAC) with breast reconstruction improves the cosmesis, body image and nipple sensation of patients, with psychosocial and aesthetic benefits [6–9].

Current guidelines suggest that the NSM approach should be limited to patients with early stage, non-high grade, and peripherally located small tumors, but these criteria have been challenged by the recognition of new, efficacious systemic therapies. An implant-based breast reconstruction (IBBR) method is used in approximately 80% of reconstructions performed after mastectomy [10, 11], and the number of immediate IBBRs using the direct-to-implant technique has also increased in Japan. This new surgical technique preserves the natural skin flap including the NAC, enabling the immediate reconstruction of the breast with a permanent implant, without the need for skin expansion with a tissue expander. Here, we review the risks and benefits of the various surgical techniques. We also analyze, retrospectively, the clinical results of IBBRs and compare the complication rates and outcomes of patients who underwent immediate (one-stage) or delayed (two-stage) IBBR following a standard total mastectomy (TM) including SSM or NSM at our hospital.

## Indications for NSM followed by IBBR

Genetic panel testing is being used increasingly to identify high-risk patients with breast cancer. In Japan, risk-reducing mastectomy as prophylactic surgery to prevent contralateral breast cancer after unilateral mastectomy for patients with BRCA gene mutations was approved by the national health insurance scheme in April, 2020. The enhanced esthetics of current surgical techniques, including NSM with breast reconstruction, make these approaches an essential option for breast cancer patients at high genetic risk. Consequently, there has been a remarkable increase in oncoplastic breast surgery supported by advances in reconstructive techniques [4, 5]. Despite the lack of prospective trial data, most international guidelines recommend NSM for risk reduction [12, 13].

Several factors should be evaluated preoperatively and intraoperatively when treating cancer in candidates for NSM, to assess the potential risk of tumor involvement of the nipple, including the tumor distance from the nipple, tumor size, and nodal status [14–16]. A positive association between tumor size and the likelihood of pathologic nipple involvement has been observed. For example, nipple involvement rates were significantly lower for smaller tumors (< 2–2.5 cm) [14, 17], and a patient with tumor distance < 1–2 cm from the nipple was approximately three times more likely to have pathologic nipple involvement [18, 19]. Thus, the practice guidelines initially suggested that patients with favorable characteristics including non-high-grade, small (< 2–2.5 cm), node-negative, and peripheral tumors ≥ 2 cm from the nipple on imaging may be suitable candidates for NSM [20]. On the other hand, SSM, which is characterized by minimal skin excision, is preferred for patients undergoing immediate IBBR. In the SSM approach, the NAC is removed, and mastectomy is performed through a small skin incision, but the flaps are preserved with overlying breast skin for the reconstruction. Therefore, if NAC involvement is found intraoperatively to be positive for malignancy on histologic examination of the nipple margins, NSM should be converted immediately to SSM [21, 22].

These criteria have been challenged by recent advances in multi-modality treatments that incorporate systemic chemotherapy, endocrine therapy, and radiotherapy, to prevent local and distant recurrences from primary breast cancer. Although there is no long-term evidence of the risk of local recurrence when the NSM approach is used, its oncologic safety is promising, with local recurrence rates comparable to those of conventional TM at 5 years [6–8, 23, 24]. Current guidelines, including those issued by the Japanese Breast Cancer Society (JBCS) and the U.S. National Comprehensive Cancer Network (NCCN), recommend simply that experienced multidisciplinary teams may consider NAC-sparing procedures for carefully selected patients with breast cancer [13]. Nevertheless, the gradually broadening indications for NSM have led to its being reconsidered for some patients who have undergone prior breast surgery and/or radiotherapy and patients with large breast tumors, ptosis, or obesity; initially considered to be contraindicated for NSM, because these factors can increase complication rates [25–29] and diminish aesthetic outcomes [30–32]. In general, the contraindications for NSM include T4 or inflammatory breast cancer, Paget’s disease, and tumors with clinical or imaging findings suggestive of extension into the NAC or patients with pathologic nipple discharge. Relative contraindications are severe and life-threatening medical comorbidities, massive obesity, and long-standing cigarette smoking [12, 13] (Table 1). Clinical studies have also shown that performing immediate IBBR based on an implant after mastectomy, including NSM, has little impact on postoperative recurrence, survival, or delayed diagnosis of recurrence [33, 34].

**Table 1:** Contraindications for implant-based breast reconstruction (IBBR) followed by nipple-sparing mastectomy (NSM)

Contraindications	T4 or inflammatory breast cancer
	Paget’s disease
	Tumors within 1 cm of the nipple or with clinical or radiologic extension into the nipple–areolar complex (NAC)
	Tumors with pathologic nipple discharge
Relative contraindications	Large breast tumors
	Ptosis
	Massive obesity
	Severe life-threatening medical comorbidity
	Long-standing cigarette smoking

Since the surgical procedure of NSM + IBBR is technically challenging, the candidates for immediate IBBR after an NSM are healthy, young non-smokers with small- or intermediate-sized and well defined breasts (B to C cup) without large ptosis, and those with biologically favorable, small, peripheral, node-negative tumors without nipple involvement [26, 34]. Other candidates include women who are undergoing prophylactic mastectomy. Bilateral reconstruction using a similar implant for the contralateral breast can also provide better symmetry and higher satisfaction [34, 35]. In addition to appropriate patient selection, to ensure the success of immediate IBBR after an NSM, radical surgery of the primary breast cancer should be prioritized for oncologic purposes over aesthetic desire.

## Surgical procedure of the NSM before IBBR

Nipple- or areolar-sparing mastectomy (NSM) should be performed by a breast surgeon, and the most common incisions used are peri- or circum-areolar, lateral, or inframammary [12, 30, 36, 37]. Incisions around or through the NAC have been reported to increase the risk of nipple necrosis and are not recommended [5, 38, 39] and radical or periareolar incisions leave visible scars on the anterior surface of the breast. Thus, lateral or inframammary skin incisions are more desirable than peri- or circum-areolar incisions for preserving blood supply to the NAC, but the choice of incision line usually depends on the breast size and the distance from the inframammary fold to the clavicle level.

**Fig. 1:**
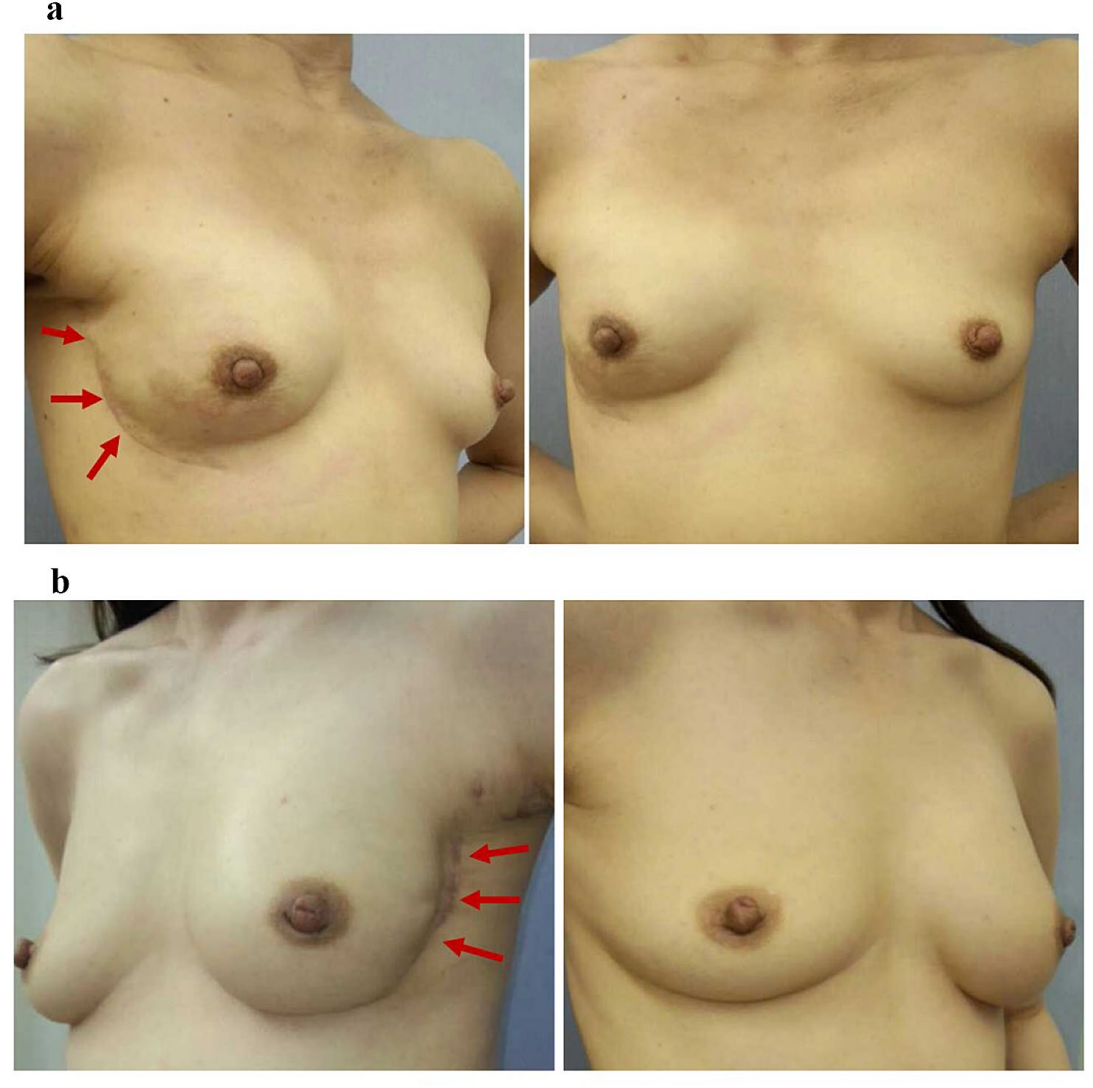
Scars (*red arrows*) of a lateral inframammary fold (IMF) incision after nipple-sparing mastectomy (NSM) followed by one-stage or two-stage implant-based breast reconstruction (IBBR). **a** One-stage IBBR. Immediate direct-to-implant reconstruction following a right NSM. **b** Two-stage IBBR. Delay in the implant reconstruction following a left NSM

**Fig. 2:**
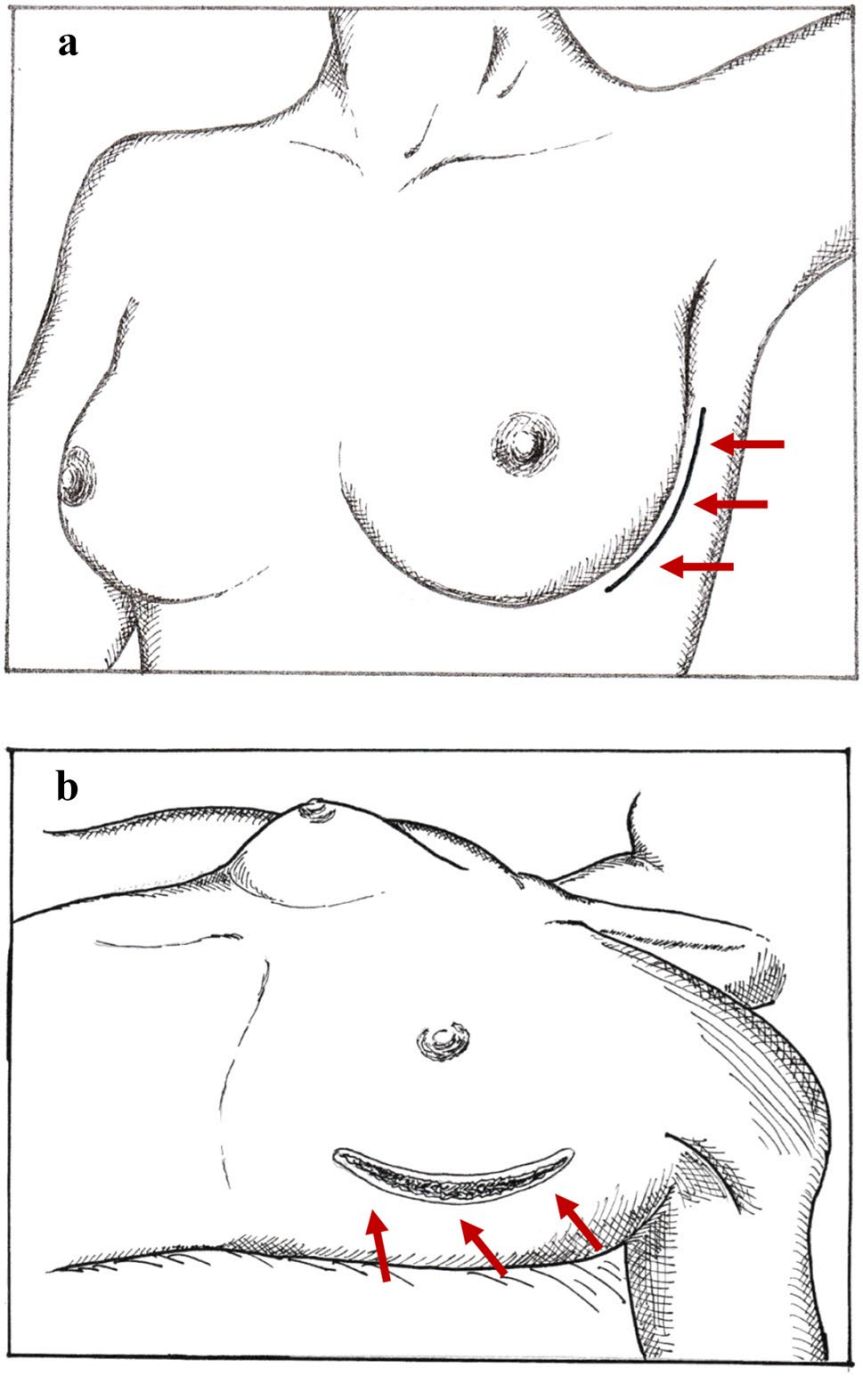
Lateral inframammary fold incision (IMF) for NSM. **a** Schema of the lateral IMF incision line (*red arrows*) (left breast). **b** Overview of the lateral IMF incision (*red arrows*) after completion of NSM and removal of the surgical specimen

Although the approach using a lateral inframammary fold (LMF) incision can make it difficult to access the upper quadrants of the breast to perform an NSM [40, 41], this is the preferred approach at our hospital, as it not only provides cosmetic exposure without leaving visible scars on the anterior surface of the breast (Fig. 1a, b), but it also allows us to perform NSM and axillary lymph node surgery through the same incision. This is important, because TM or NSM combined with sentinel node biopsy and/or axillary lymph node dissection is the standard procedure for breast cancer surgery (Fig. 2b). The LMF incision is made along the curvilinear skin crease, starting from the lateral site, around the 3 o’clock position for the left breast or the 9 o’clock position for the right breast, and extending inferiorly to near the 6 o’clock position (Fig. 2a, b).

NSM should leave the dermis and epidermis of the NAC at the level of the subcutaneous fat layer to preserve an NAC flap that is ≥ 3 mm thick. It should also remove the major ducts using intraoperative frozen sections to identify the clear surgical margin of the sub-areolar region. The resected sub-areolar nipple ductal tissue on the surgical breast specimen from an NSM should be marked with a suture for subsequent pathologic examination [22, 36, 42]. At our hospital, the sub-areolar nipple duct margin is evaluated by intraoperative frozen section as well as postoperative permanent specimens. If the frozen section and the permanent pathology reveal in situ or invasive carcinoma of the ductal tissue, the carcinoma and NAC will be removed during the same operation and/or in a second surgical procedure.

After the entire resected breast specimen of the NSM is removed, the viability of the NAC and skin flap will be assessed by evaluating the color, temperature, and dermal blood flow of the skin flap. The viability of the local blood supply of a skin flap after a TM or NSM can also be assessed intraoperatively using a fluorescein imaging system with indocyanine green (ICG) dye, which can also detect axillary sentinel lymph nodes [43–45]. If the NAC and skin flap are both viable, breast reconstruction can be performed by reconstructive plastic surgeons using either the immediate direct-to-implant technique as one-stage reconstruction, or by tissue expanders that require a change of the expander to a definitive implant, as two-stage reconstruction.

## Complications of NSM followed by IBBR

### One-stage and two-stage IBBR

An IBBR is usually well tolerated and safe, with low rates of major and minor complications. The typical complications include skin necrosis, infection, infection requiring implant removal, and hematoma/seroma [33]. Several studies have shown that immediate reconstruction (one-stage IBBR) has the same postoperative complications as tissue expander/implant-based breast reconstruction (two-stage IBBR) [46]. However, historical data suggest that two-stage IBBR using the sub-muscular implant space is reliable, safe, and effective [47–49], and that the complication rates of one-stage IBBR tend to be higher [50–52].

A recent meta-analysis found no significant difference between one-stage IBBR and two-stage IBBR in terms of complication rates of infection, seroma, hematoma, and capsule contracture, but the incidences of flap necrosis, reoperation, and implant loss were significantly higher after one-stage IBBR [46]. Other studies show that the postoperative complication rate for NSM is approximately 20–30% and that the rate of complications requiring treatment remains at approximately 10–12% [11, 26, 38, 53]. Moreover, the reported rate of complications involving an NSM followed by one-stage or two-stage IBBR ranges from 1.5 to 9% for surgical-site infection [38, 54–57], 1–5% for seroma requiring treatment [56–58], 1–3% for hematoma [56, 57], 4–20% for skin flap ischemia, and 3–12% for necrosis [38, 55, 56, 58]. The incidences of reversible ischemia and/or superficial epidermolysis of the nipple lesion range from 6 to 13% [55, 57, 58], and that of NAC necrosis resulting in nipple loss ranges from 1–5% [4, 26, 38, 55, 59]. Skin incisions for expander/implant placement away from the areola were reported to be associated with fewer ischemic complications of the NAC [11, 53, 60].

NSM followed by immediate IBBR is still a technically challenging surgical procedure, but several research groups have described individual and/or team learning curves for NSM based on patient selection, surgical judgment, technical expertise, and perioperative management [5, 53, 54].

### Chemotherapy and radiotherapy

Adjuvant chemotherapy has been reported to increase the overall complication rate to 27–30% [61–63], whereas neoadjuvant chemotherapy has been reported to increase it to within the range of 15–33% [64, 65]. Moreover, definitive reconstruction failed in 38% of patients who received neoadjuvant chemotherapy because of infection or extrusion [64]. The safety of neoadjuvant chemotherapy remains a subject of controversy, although two studies reported that neoadjuvant chemotherapy did not increase the complication rate and was safe for IBBR [66, 67]. A systematic review found that even one-stage IBBR does not necessarily delay the start of adjuvant chemotherapy to a clinically relevant extent, suggesting that one- or two-stage IBBR is an effective option for patients with early stage breast cancer [68, 69]. On the other hand, although complications from a TM including SSM or NSM followed by one- or two-stage IBBR may delay the timing of postoperative adjuvant chemotherapy when initiating treatment, no impact of neo- or adjuvant chemotherapy on oncologic and cosmetic outcomes was observed [66, 67].

Patients with ipsilateral loco-regional recurrence after prior breast breast-conserving surgery plus radiotherapy are at higher risk of complications following TM including SSM or NSM with the one- or two-stage IBBR approach [54]. In a series of patients who had undergone prior whole-breast radiation and subsequent NSM followed by IBBR, the rate of infection was 20%, that of expander loss at the first stage was 15%, while that of implant loss was 5% [70]. A retrospective analysis revealed that the substantial rate of early postoperative complications in NSM patients who had received prior radiotherapy included an 18.8% rate of infection, necrosis, and hematoma requiring reoperation, a 7.2% rate of nipple necrosis, and a 4.3% rate of nipple loss [26]. In contrast, the rate of implant loss and surgical-site infection were approximately 15–22% and 9–31%, respectively, for patients who received postoperative radiotherapy [70, 71].

### Our experience

We analyzed 71 patients who underwent TM as SSM or NSM, followed by one- or two-stage IBBR, at our hospital after 2013 and compared their clinical features and complication rates. As shown in Table 2, early stage (0–II) disease with minimal lymph node metastasis was diagnosed in all patients. Thirty-seven patients underwent TM, including 5 who underwent SSM, followed by two-stage IBBR; 18 who underwent NSM followed by two-stage IBBR; and 16 who underwent NSM followed by one-stage IBBR. The operation times were 236, 247.2, and 321 min, and the mean surgical blood loss was 60.4, 88.8, and 138 ml, respectively. Axillary surgery consisted of a sentinel lymph node biopsy in 31, 13, and 14 patients, respectively, and a sentinel node biopsy and/or axillary node dissection in 4, 4, and 2 patients, respectively. Neo- or adjuvant chemotherapy was given to 16 (43.2%), 8 (44.4%), and 6 (37.5%) patients, respectively, and adjuvant endocrine therapy was given to 26 (70.3%), 11 (61.1%), and 7 (43.8%) patients, respectively.

**Table 2:** Clinical characteristics of the patients

	TM + TE (two-stage IBBR)	NSM + TE (two-stage IBBR)	NSM + direct-to-implant (one-stage IBBR)
No. of patients	37	18	16
Median age, yrs (range)	49 (35–76)	47 (30–61)	50 (42–74)
Pathological stage (%)			
Stage 0 (DCIS/LCIS) cases	5 (13.5)	8 (44.4)	4 (25)
Stage I cases	22 (59.5)	8 (44.4)	6 (37.5)
Stage II cases	10 (27)	2 (11.2)	6 (37.5)
Biologic subtype (%)			
Luminal cases	27 (72.9)	15 (83.2)	14 (87.5)
HER2-positive cases	8 (21.6)	2 (11.2)	1 (6.25)
Triple-negative cases	2 (5.5)	1 (5.6)	1 (6.25)
Operation time, mean	236.0 min.	247.2 min.	321.0 min.
Sentinel lymph node biopsy (%)	31 (83.8)	13 (72.2)	14 (87.5)
Axillary lymph node dissection* (%)	4 (10.8)	4 (22.2)	2 (11.8)
Blood loss, mean ml	60.4	88.8	138.0
Hospitalization, mean days	16.7	15.2	14.0
Chemotherapy (%)	16 (43.2)	8 (44.4)	6 (37.5)
Neoadjuvant/adjuvant	(2/14)	(1/7)	(1/6)
Adjuvant endocrine therapy (%)	26 (70.3)	11 (61.1)	7 (43.8)

TM: total mastectomy including skin-sparing mastectomy (SSM); NSM: nipple-sparing mastectomy; TE: tissue expander; IBBR: implant based breast reconstruction

*All cases: pN1a

The complications in these three groups included infection (2.7%, 11.1%, and 6.3%), seroma/hematoma (0%, 5.6%, and 6.3%), flap necrosis (2.7%, 11.1%, and 12.5%), and loss of tissue expander or implant (2.7%, 5.6%, and 6.3%), respectively (Table 3). Specifically, one patient from the NSM + two-stage IBBR group, who had received radiotherapy after initial breast-conserving surgery, suffered nipple necrosis, and the nipple was lost in one patient from the NSM + one-stage IBBR group who was a heavy smoker (Table 2). The total complication rates were 8.1% for the TM + two-stage IBBR group, 38.9% for the NSM + two-stage IBBR group, and 31.3% for the NSM + one-stage IBBR group (Table 3).

**Table 3:** Postoperative complications expressed as percentages (total number of each complication)

	TM + TE (two-stage IBBR)	NSM + TE (two-stage IBBR)	NSM + direct-to implant (one-stage IBBR)
Total complication rate	8.1 (2)	38.9 (7)	31.3 (5)
Infection	2.7 (1)	11.1 (2)	6.3 (1)
Seroma/hematoma	0	5.6 (1)	6.3 (1)
Flap ischemia/necrosis	2.7 (1)	11.1 (2)	12.5 (2)
TE/implant loss	2.7 (1)	5.6 (1*)	6.3 (1^#^)
Nipple necrosis/loss	–	5.6 (1)	6.3 (1)

*The patient underwent prior radiotherapy after initial breast-conserving surgery

^#^The patient was a heavy smoker

These findings are consistent with those of previous studies and indicate that one-stage IBRR tended to increase the operating time and blood loss vs. two-stage IBRR and that there was no significant difference in the complication rate between one-stage and two-stage IBBR. In contrast, the complication rate might be higher when NSM is followed by IBBR than when TM with or without SSM is followed by IBBR.

## Oncological outcomes of NSM followed by IBBR

The major oncologic concern about NSM followed by IBBR is the possibility that residues of primary cancer may be left in the breast tissue behind the NAC, which is usually preserved for blood supply. However, a review of ten studies of 1148 patients who underwent conventional TM indicated that the loco-regional recurrence rate was 2.8%, whereas that after NSM was 4.4% and 7.8% for patients with invasive breast cancer and ductal carcinoma in situ (DCIS), respectively [72, 73]. The 5-year rates of local recurrence of invasive ductal carcinoma (IDC) and DCIS in the NAC were 0.8% and 2.9%, respectively, which are lower than those of 3.6% and 4.9% in the chest wall [34, 72]. More recent single-institution studies, including our own retrospective analyses, clarified that the loco-regional recurrence rates at NAC sites and non-NAC sites ranged from 0% to 3.7% and from 0% to 8.2%, respectively (Table 4).

**Table 4:** Oncologic outcomes of nipple-sparing mastectomy followed by implant-based breast construction

Data	Year of publication	No. of breasts operated on	Median follow-up in months	Local regional recurrence rate (%)	
				NAC*	Not NAC (e.g., skin flap, chest wall)
Kim [81]	2020	251	68 (mean)	0.4	4
Krajewski [5]	2015	236	24	0	1.7
Eisenberg [80]	2014	208	33 (mean)	0.5	0.5
Sakurai [24]	2013	788	78	3.7	8.2
Coopey [4]	2013	315	22	0	2.6
Peled [38]	2012	412	28	0	2
Spear [83]	2011	49	30 (mean)	0	0
Kim [21]	2010	152	60	1.3	2
Our own^#^	–	34	38	0	0

*At the nipple–areolar complex

^**#**^Data included NSM followed by one-stage or two-stage reconstruction

The results of our investigations are consistent with those of studies showing that loco-regional recurrence is less likely in the NAC regardless of whether patients undergo one-stage or two-stage IBBR following NSM. One of our 71 patients who underwent neoadjuvant chemotherapy was found to have distant lymph node metastases at the 24-month follow-up after NSM + two-stage IBBR; however, at the median 38-month follow-up, no loco-regional recurrence at the NAC or other distant recurrence were detected in any of the patients, irrespective of whether they underwent TM, NSM + one-stage IBBR, or NSM + two-stage IBBR.

## Implant-related systemic disease

Breast implant-associated anaplastic large cell lymphoma (BIA-ALCL) is a rare disease that may occur in women who have had an implant inserted as part of the reconstructive operation. An increased risk of BIA-ALCL in women with breast implants was first described in 2006. A significant association between breast BIA-ALCL and textured implants has been noted, as IBBR remains a common method of breast reconstruction worldwide for patients with breast cancer as well as for women who undergo breast augmentation with silicone implants. BIA-ALCL is an anaplastic lymphoma kinase (ALK)-negative and CD30-positive T cell lymphoma that arises in either the fluid or capsule surrounding the implant. The first case of BIA-ALCL in Japan was also recently reported [74, 75].

Other systemic diseases are occasionally observed after IBBR, and several studies have described an association between breast implants and connective tissue disorders, immune dysregulation, cancer (including breast cancer), and neurological diseases [76–78]. Some cases were recorded as alleged breast implant-related deaths [79]. An implant used for cosmetic breast augmentation may interfere with the detection of breast cancer and impact the patient’s survival [80].

## Limitations

This review has several limitations. First, most of the studies we reviewed were performed at single institutions, and thus the data may tend to be biased. Second, we lacked the long-term maintenance data necessary to identify the rate of complications and disadvantages related to IBBR, including data about ruptures, leaks, the symmetry of implants in unilateral reconstruction, and capsular contracture.

## Conclusion

With a better understanding of tumor biology and the use of increasingly effective neoadjuvant and adjuvant therapies, the oncologic outcome data are encouraging. There are currently low loco-regional recurrence rates after the surgical treatment of breast cancer using NSM followed by IBBR. The NSM technique is an attractive procedure both for high-risk patients with BRCA mutations and for use in conjunction with contralateral prophylactic mastectomy for patients with unilateral breast cancer. The NSM may become a standard surgical procedure, and its complications are comparable to those of the traditional TM. Nevertheless, it is important to inform patients appropriately about the oncologic safety, complications, and cosmetic outcomes of the various options. The choice of whether to perform an NSM or TM followed by IBBR should be individualized in the appropriate setting, as well as with careful preoperative management in accordance with the patient’s preferences, including considerations of patient satisfaction and reasonable expectations.

## References

[1] International Agency for Research on Cancer. Cancer Today: https://gco.iarc.fr/today/online-analysis-multi-bars?v=2018&mode=cancer&mode_population/. Accessed June 2, 2020.

[2] National Breast Cancer Patient Registration Survey Report (2016) (in Japanese): https://memberpage.jbcs.gr.jp/C52/menu_details/28. Accessed June 2, 2020.

[3] National Cancer Center Japan. Latest Cancer Statistics (in Japanese): https://ganjoho.jp/reg_stat/statistics/stat/summary.html. Accessed June 2, 2020.

[4] Coopey SB, Tang R, Lei L, Freer PE, Kansal K, Colwell AS (2013). Increasing eligibility for nipple-sparing mastectomy. Ann Surg Oncol.

[5] Krajewski AC, Boughey JC, Degnim AC, Jakub JW, Jacobson SR, Hoskin TL (2015). Expanded indications and improved outcomes for nipple-sparing mastectomy over time. Ann Surg Oncol.

[6] Gerber B, Krause A, Dieterich M, Kundt G, Reimer T (2009). The oncological safety of skin sparing mastectomy with conservation of the nipple-areola complex and autologous reconstruction: an extended follow-up study. Ann Surg.

[7] Didier F, Radice D, Gandini S, Bedolis R, Rotmensz N, Maldifassi A (2009). Does nipple preservation in mastectomy improve satisfaction with cosmetic results, psychological adjustment, body image and sexuality?. Breast Cancer Res Treat.

[8] Metcalfe KA, Cil TD, Semple JL, Li LD, Bagher S, Zhong T (2015). Long-term psychosocial functioning in women with bilateral prophylactic mastectomy: does preservation of the nipple-areolar complex make a difference?. Ann Surg Oncol.

[9] van Verschuer VM, Mureau MA, Gopie JP, Vos EL, Verhoef C, Menke-Pluijmers MB (2016). Patient satisfaction and nipple-areola sensitivity after bilateral prophylactic mastectomy and immediate implant breast reconstruction in a high breast cancer risk population: nipple-sparing mastectomy versus skin-sparing mastectomy. Ann Plast Surg.

[10] Albornoz CR, Bach PB, Mehrara BJ, Disa JJ, Pusic AL, McCarthy CM (2013). A paradigm shift in US Breast reconstruction: increasing implant rates. Plast Reconstr Surg.

[11] Endara M, Chen D, Verma K, Nahabedian MY, Spear SL (2013). Breast reconstruction following nipple-sparing mastectomy: a systematic review of the literature with pooled analysis. Plast Reconstr Surg.

[12] Weber WP, Haug M, Kurzeder C, Bjelic-Radisic V, Koller R, Reitsamer R (2018). Oncoplastic breast consortium consensus conference on nipple-sparing mastectomy. Breast Cancer Res Treat.

[13] NCCN Clinical Practice Guidelines: Breast Cancer [Internet]. 2020. v4. https://www.nccn.org/professionals/physician_gls/pdf/breast.pdf. Accessed June 2, 2020.

[14] Brachtel EF, Rusby JE, Michaelson JS, Chen LL, Muzikansky A, Smith BL (2009). Occult nipple involvement in breast cancer: clinicopathologic findings in 316 consecutive mastectomy specimens. J Clin Oncol.

[15] Cense HA, Rutgers EJ, Lopes Cardozo M, Van Lanschot JJ (2001). Nipple-sparing mastectomy in breast cancer: a viable option?. Eur J Surg Oncol.

[16] Laronga C, Kemp B, Johnston D, Robb GL, Singletary SE (1999). The incidence of occult nipple-areola complex involvement in breast cancer patients receiving a skin-sparing mastectomy. Ann Surg Oncol.

[17] Steen ST, Chung AP, Han SH, Vinstein AL, Yoon JL, Giuliano AE (2013). Predicting nipple-areolar involvement using preoperative breast MRI and primary tumor characteristics. Ann Surg Oncol.

[18] Billar JA, Dueck AC, Gray RJ, Wasif N, Pockaj BA (2011). Preoperative predictors of nipple-areola complex involvement for patients undergoing mastectomy for breast cancer. Ann Surg Oncol.

[19] Fortunato L, Loreti A, Andrich R, Costarelli L, Amini M, Farina M (2013). When mastectomy is needed: is the nipple-sparing procedure a new standard with very few contraindications?. J Surg Oncol.

[20] Laronga C, Smith P (2014). Nipple-sparing mastectomy: an oncologic and cosmetic perspective. Surg Oncol Clin N Am.

[21] Toth BA, Lappert P (1991). Modified skin incisions for mastectomy: the need for plastic surgical input in preoperative planning. Plast Reconstr Surg.

[22] Boneti C, Yuen J, Santiago C, Diaz Z, Robertson Y, Korourian S (2011). Oncologic safety of nipple skin-sparing or total skin-sparing mastectomies with immediate reconstruction. J Am Coll Surg.

[23] Kim HJ, Park EH, Lim WS, Seo JY, Koh BS, Lee TJ (2010). Nipple areola skin-sparing mastectomy with immediate transverse rectus abdominis musculocutaneous flap reconstruction is an oncologically safe procedure: a single center study. Ann Surg.

[24] Sakurai T, Zhang N, Suzuma T, Umemura T, Yoshimura G, Sakurai T (2013). Long-term follow-up of nipple-sparing mastectomy without radiotherapy: a single center study at a Japanese institution. Med Oncol.

[25] Frederick MJ, Lin AM, Neuman R, Smith BL, Austen WG, Colwell AS (2015). Nipple-sparing mastectomy in patients with previous breast surgery: comparative analysis of 775 immediate breast reconstructions. Plast Reconstr Surg.

[26] Tang R, Coopey SB, Colwell AS, Specht MC, Gadd MA, Kansal K (2015). Nipple-sparing mastectomy in irradiated breasts: selecting patients to minimize complications. Ann Surg Oncol.

[27] Hieken TJ, Boolbol SK, Dietz JR (2016). Nipple-sparing mastectomy: indications, contraindications, risks, benefits, and techniques. Ann Surg Oncol.

[28] Murphy BL, Boughey JC, Hieken TJ (2017). Nipple-sparing mastectomy for the management of recurrent breast cancer. Clin Breast Cancer.

[29] Murphy BL, Hoskin TL, Boughey JC, Degnim AC, Jakub JW, Krajewski AC (2017). Outcomes and feasibility of nipple-sparing mastectomy for node-positive breast cancer patients. Am J Surg.

[30] Stolier AJ, Sullivan SK, Dellacroce FJ (2008). Technical considerations in nipple-sparing mastectomy: 82 consecutive cases without necrosis. Ann Surg Oncol.

[31] Wagner JL, Fearmonti R, Hunt KK, Hwang RF, Meric-Bernstam F, Kuerer HM (2012). Prospective evaluation of the nipple-areola complex sparing mastectomy for risk reduction and for early-stage breast cancer. Ann Surg Oncol.

[32] Colwell AS, Tessler O, Lin AM, Liao E, Winograd J, Cetrulo CL (2014). Breast reconstruction following nipple-sparing mastectomy: predictors of complications, reconstruction outcomes, and 5-year trends. Plast Reconstr Surg.

[33] McCarthy CM, Mehrara BJ, Riedel E, Davidge K, Hinson A, Disa JJ (2008). Predicting complications following expander/implant breast reconstruction: an outcomes analysis based on preoperative clinical risk. Plast Reconstr Surg.

[34] Jay R. Harris MEL, Monica M, Kent Osborne C (2014) Diseases of the Breast. 5th edn. Lippincott Williams & Wilkins, vol 504-09, pp 536–540.

[35] Shaikh-Naidu N, Preminger BA, Rogers K, Messina P, Gayle LB (2004). Determinants of aesthetic satisfaction following TRAM and implant breast reconstruction. Ann Plast Surg.

[36] Crowe JP, Kim JA, Yetman R, Banbury J, Patrick RJ, Baynes D (2004). Nipple-sparing mastectomy: technique and results of 54 procedures. Arch Surg.

[37] Garcia-Etienne CA, Cody Iii HS, Disa JJ, Cordeiro P, Sacchini V (2009). Nipple-sparing mastectomy: Initial experience at the Memorial Sloan-Kettering Cancer Center and a comprehensive review of literature. Breast J.

[38] Warren Peled A, Foster RD, Stover AC, Itakura K, Ewing CA, Alvarado M (2012). Outcomes after total skin-sparing mastectomy and immediate reconstruction in 657 breasts. Ann Surg Oncol.

[39] Wijayanayagam A, Kumar AS, Foster RD, Esserman LJ (2008). Optimizing the total skin-sparing mastectomy. Arch Surg.

[40] Rawlani V, Fiuk J, Johnson SA, Buck DW, Hirsch E, Hansen N (2011). The effect of incision choice on outcomes of nipple-sparing mastectomy reconstruction. Can J Plast Surg.

[41] Blechman KM, Karp NS, Levovitz C, Guth AA, Axelrod DM, Shapiro RL (2013). The lateral inframammary fold incision for nipple-sparing mastectomy: outcomes from over 50 immediate implant-based breast reconstructions. Breast J.

[42] Bertozzi N, Pesce M, Santi P, Raposio E (2017). One-stage immediate breast reconstruction: a concise review. Biomed Res Int.

[43] Singer R, Lewis CM, Franklin JD, Lynch JB (1978). Fluorescein test for prediction of flap viability during breast reconstructions. Plast Reconstr Surg.

[44] Losken A, Styblo TM, Schaefer TG, Carlson GW (2008). The use of fluorescein dye as a predictor of mastectomy skin flap viability following autologous tissue reconstruction. Ann Plast Surg.

[45] Toh U, Iwakuma N, Mishima M, Okabe M, Nakagawa S, Akagi Y (2015). Navigation surgery for intraoperative sentinel lymph node detection using Indocyanine green (ICG) fluorescence real-time imaging in breast cancer. Breast Cancer Res Treat.

[46] Basta MN, Gerety PA, Serletti JM, Kovach SJ, Fischer JP (2015). A systematic review and head-to-head meta-analysis of outcomes following direct-to-implant versus conventional two-stage implant reconstruction. Plast Reconstr Surg.

[47] Cordeiro PG, McCarthy CM (2006). A single surgeon’s 12-year experience with tissue expander/implant breast reconstruction: part I. A prospective analysis of early complications. Plast Reconstr Surg.

[48] Cordeiro PG, McCarthy CM (2006). A single surgeon’s 12-year experience with tissue expander/implant breast reconstruction: part II. An analysis of long-term complications, aesthetic outcomes, and patient satisfaction. Plast Reconstr Surg.

[49] Lennox PA, Bovill ES, Macadam SA (2017). Evidence-based medicine: alloplastic breast reconstruction. Plast Reconstr Surg.

[50] Hvilsom GB, Friis S, Frederiksen K, Steding-Jessen M, Henriksen TF, Lipworth L (2011). The clinical course of immediate breast implant reconstruction after breast cancer. Acta Oncol.

[51] Fischer JP, Wes AM, Tuggle CT, Serletti JM, Wu LC (2013). Risk analysis of early implant loss after immediate breast reconstruction: a review of 14,585 patients. J Am Coll Surg.

[52] Pinsolle V, Grinfeder C, Mathoulin-Pelissier S, Faucher A (2006). Complications analysis of 266 immediate breast reconstructions. J Plast Reconstr Aesthet Surg.

[53] Wang F, Peled AW, Garwood E, Fiscalini AS, Sbitany H, Foster RD (2014). Total skin-sparing mastectomy and immediate breast reconstruction: an evolution of technique and assessment of outcomes. Ann Surg Oncol.

[54] Young WA, Degnim AC, Hoskin TL, Jakub JW, Nguyen MD, Tran NV (2019). Outcomes of > 1300 nipple-sparing mastectomies with immediate reconstruction: the impact of expanding indications on complications. Ann Surg Oncol.

[55] Donovan CA, Harit AP, Chung A, Bao J, Giuliano AE, Amersi F (2016). Oncological and surgical outcomes after nipple-sparing mastectomy: do incisions matter?. Ann Surg Oncol.

[56] Frey JD, Choi M, Salibian AA, Karp NS (2017). Comparison of outcomes with tissue expander, immediate implant, and autologous breast reconstruction in greater than 1000 nipple-sparing mastectomies. Plast Reconstr Surg.

[57] Mitchell SD, Willey SC, Beitsch P, Feldman S (2018). Evidence based outcomes of the American Society of Breast Surgeons Nipple Sparing Mastectomy Registry. Gland Surg.

[58] De Vita R, Zoccali G, Buccheri EM, Costantini M, Botti C, Pozzi M (2017). Outcome evaluation after 2023 nipple-sparing mastectomies: our experience. Plast Reconstr Surg.

[59] Galimberti V, Morigi C, Bagnardi V, Corso G, Vicini E, Fontana SKR (2018). Oncological outcomes of nipple-sparing mastectomy: a single-center experience of 1989 patients. Ann Surg Oncol.

[60] Petit JY, Veronesi U, Lohsiriwat V, Rey P, Curigliano G, Martella S (2011). Nipple-sparing mastectomy—is it worth the risk?. Nat Rev Clin Oncol.

[61] Eck DL, McLaughlin SA, Terkonda SP, Rawal B, Perdikis G (2015). Effects of immediate reconstruction on adjuvant chemotherapy in breast cancer patients. Ann Plast Surg.

[62] Furey PC, Macgillivray DC, Castiglione CL, Allen L (1994). Wound complications in patients receiving adjuvant chemotherapy after mastectomy and immediate breast reconstruction for breast cancer. J Surg Oncol.

[63] O’Brien W, Hasselgren PO, Hummel RP, Coith R, Hyams D, Kurtzman L (1993). Comparison of postoperative wound complications and early cancer recurrence between patients undergoing mastectomy with or without immediate breast reconstruction. Am J Surg.

[64] Mitchem J, Herrmann D, Margenthaler JA, Aft RL (2008). Impact of neoadjuvant chemotherapy on rate of tissue expander/implant loss and progression to successful breast reconstruction following mastectomy. Am J Surg.

[65] Donker M, Hage JJ, Woerdeman LA, Rutgers EJ, Sonke GS, Vrancken Peeters MJ (2012). Surgical complications of skin sparing mastectomy and immediate prosthetic reconstruction after neoadjuvant chemotherapy for invasive breast cancer. Eur J Surg Oncol.

[66] Xavier Harmeling J, Kouwenberg CA, Bijlard E, Burger KN, Jager A, Mureau MA (2015). The effect of immediate breast reconstruction on the timing of adjuvant chemotherapy: a systematic review. Breast Cancer Res Treat.

[67] Song J, Zhang X, Liu Q, Peng J, Liang X, Shen Y (2014). Impact of neoadjuvant chemotherapy on immediate breast reconstruction: a meta-analysis. PLoS ONE.

[68] Cohen O, Lam G, Choi M, Karp N, Ceradini D (2017). Does the timing of chemotherapy affect post-mastectomy breast reconstruction complications?. Clin Breast Cancer.

[69] O’Connell RL, Rattay T, Dave RV, Trickey A, Skillman J, Barnes NLP (2019). The impact of immediate breast reconstruction on the time to delivery of adjuvant therapy: the iBRA-2 Study. Br J Cancer.

[70] Peled AW, Sears M, Wang F, Foster RD, Alvarado M, Wong J (2018). Complications after total skin-sparing mastectomy and expander-implant reconstruction: effects of radiation therapy on the stages of reconstruction. Ann Plast Surg.

[71] Jagsi R, Jiang J, Momoh AO, Alderman A, Giordano SH, Buchholz TA (2016). Complications after mastectomy and immediate breast reconstruction for breast cancer: a claims-based analysis. Ann Surg.

[72] Petit JY, Veronesi U, Orecchia R (2009). Nipple-sparing mastectomy with nipple areola intraoperative radiotherapy: one thousand and one cases of a five years experience at the European Institute of Oncology in Milan (EIO). Breast Cancer Res Treat.

[73] Piper M, Peled AW, Foster RD, Moore DH, Esserman LJ (2013). Total skin-sparing mastectomy: a systematic review of oncologic outcomes and postoperative complications. Ann Plast Surg.

[74] O’Neill AC, Zhong T, Hofer SOP (2017). Implications of breast implant-associated anaplastic large cell lymphoma (BIA-ALCL) for breast cancer reconstruction: an update for surgical oncologists. Ann Surg Oncol.

[75] Ohishi Y, Mitsuda A, Ejima K, Morizono H, Yano T, Yokoyama M (2020). Breast implant-associated anaplastic large-cell lymphoma: first case detected in a Japanese breast cancer patient. Breast Cancer.

[76] Janowsky EC, Kupper LL, Hulka BS (2000). Meta-analyses of the relation between silicone breast implants and the risk of connective-tissue diseases. N Engl J Med.

[77] Singh N, Picha GJ, Hardas B, Schumacher A, Murphy DK (2017). Five-year safety data for more than 55,000 subjects following breast implantation: comparison of rare adverse event rates with silicone implants versus national norms and saline implants. Plast Reconstr Surg.

[78] Balk EM, Earley A, Avendano EA, Raman G (2016). Long-term health outcomes in women with silicone gel breast implants: a systematic review. Ann Intern Med.

[79] Abi-Rafeh J, Safran T, Alhalabi B, Dionisopoulos T (2019). Death by implants: a critical analysis of the FDAMAUDE Database on breast implant-related mortality. Plast Reconstr Surg Glob Open..

[80] Lavigne E, Holowaty EJ, Pan SY, Villeneuve PJ, Johnson KC, Fergusson DA (2013). Breast cancer detection and survival among women with cosmetic breast implants: systematic review and meta-analysis of observational studies. BMJ.

[81] Kim S, Lee S, Bae Y, Lee S (2020). Nipple-sparing mastectomy for breast cancer close to the nipple: a single institution’s 11-year experience. Breast Cancer.

[82] Eisenberg RE, Chan JS, Swistel AJ, Hoda SA (2014). Pathological evaluation of nipple-sparing mastectomies with emphasis on occult nipple involvement: the Weill-Cornell experience with 325 cases. Breast J.

[83] Spear SL, Willey SC, Feldman ED, Cocilovo C, Sidawy M, Al-Attar A (2011). Nipple-sparing mastectomy for prophylactic and therapeutic indications. Plast Reconstr Surg.

